# Cryoballoon-Induced Circumferential Pulmonary Vein Fibrosis, Assessed by Late Gadolinium-Enhancement Cardiac Magnetic Resonance Imaging, and Its Correlation with Clinical Atrial Fibrillation Recurrence

**DOI:** 10.3390/jcm12062442

**Published:** 2023-03-22

**Authors:** Moshe Rav Acha, Oholi Tovia-Brodie, Yoav Michowitz, Feras Bayya, Fauzi F. Shaheen, Shalom Abuhatzera, Aharon Medina, Michael Glikson, Arik Wolak

**Affiliations:** 1 Jesselson Integrated Heart Center, Shaare Zedek Hospital, Jerusalem 9112102, Israel; 2 Faculty of Medicine, Hebrew University, Jerusalem 9112102, Israel; 3 Shamir Medical Center, Cardiology Department, Be’er-Yaakov 7033001, Israel; 4 Faculty of Medicine, Tel Aviv University, Tel Aviv 900050, Israel

**Keywords:** MRI, cryoballoon, ablation-induced, fibrosis, AF, recurrence

## Abstract

Background: Prior studies evaluating post-atrial fibrillation (AF) ablation pulmonary vein (PV) ostial gaps via magnetic resonance imaging (MRI) have shown circumferential PV fibrosis in a minority of patients, and their correlation with AF recurrence was weak. These studies were mostly based on radio-frequency AF ablations. Aim: We aimed to assess cryoballoon ablation-induced PV fibrosis via MRI and its correlation with AF recurrence. Methods and Results: This was a prospective study of consecutive patients with symptomatic AF who underwent pre- and post-ablation MRI to assess baseline and ablation-induced fibrosis, respectively. Post-ablation PV gaps were assessed by new semi-quantitative visual analysis assisted by computerized ADAS analysis. AF recurrence monitored via multiple ECGs and event monitoring at 6 and 12 months post ablation. Nineteen patients with 80 PVs were included, age 56 ± 11, with paroxysmal and persistent AF in 17/19 and 2/19 patients, respectively. Baseline MRI showed minimal LA fibrosis. All patients underwent successful cryoballoon PV electrical isolation. Post-ablation MRI revealed circumferential PV fibrosis among 63/80 (78.8%) PVs and partial fibrosis with major gaps among 17/80 (21.2%) PVs. AF recurred within one year in 5/9 (55.5%) patients with partial PV fibrosis, while no AF recurred among the 10 patients in whom all PVs had circumferential fibrosis (*p* < 0.01). Similarly, there were significantly more PVs without circumferential fibrosis (due to major gaps) among patients with AF recurrence as compared with patients without AF recurrence (42.9% vs. 13.5%; *p* < 0.01). Conclusion: Cryoballoon AF ablation results in circumferential PV fibrosis in the majority of PVs, as assessed by a new clinically relevant MRI-LGE analysis. Significant correlation was found between major PV gaps on post-ablation MRI and AF recurrence, suggesting that MRI might have the ability to predict AF recurrence.

## 1. Introduction

Assessment and quantification of baseline left atrial (LA) fibrosis via late gadolinium enhancement (LGE) on cardiac magnetic resonance imaging (MRI) was found to predict AF ablation success, namely post-ablation AF recurrence [1,2,3]. Multiple studies have shown MRI could precisely identify atrial fibrosis, which is the final common pathway of many functional and structural abnormalities known to contribute to AF persistence [1,2,3]. Since the pivot of AF ablation is PV electrical isolation (PVI), the presence of a significant extra-PV fibrosis as seen on pre-ablation MRI could explain AF recurrence despite successful PVI [1,2,4].

In contrast with the consensus regarding the feasibility of pre-ablation MRI to assess baseline LA fibrosis, the ability of post-ablation MRI to evaluate ablation-induced LA lesions, with emphasis on ablation-induced PV ostial fibrosis, is up for debate. Some argue that post-ablation MRI could reliably detect ablation-induced fibrosis both at LA body and PV ostia levels, showing good correlation between PV gaps found on post-ablation MRI and those found on electro-anatomic mapping (EAM) done before the redo ablation [5,6,7]. Many others, however, suggest a modest or even poor correlation between post-ablation MRI and redo EAM regarding ablation-induced PV ostial gaps [8,9,10,11]. 

Furthermore, the correlation between post-ablation MRI PV gaps and AF recurrence is doubtful. In many studies, only a minority of PVs had complete circumferential fibrosis on post-ablation MRI despite undergoing successful PVI, and the correlation between post-ablation PV circumferential fibrosis and AF recurrence was weak and non-significant. For example, among 177 patients who underwent MRI three months post AF ablation, only 12/177 (7%) patients had a complete circumferential fibrosis of all PVs [9], and the presence of circumferential PV fibrosis was not associated with AF recurrence [9]. In another study [10], a modest correlation was found between the presence of circumferential PV fibrosis on an MRI performed one to two months post AF ablation and AF recurrence. However, even among those patients without AF recurrence, only 55% of PVs had circumferential fibrosis [10]. 

Importantly, the vast majority of AF ablations evaluated within the above studies comprised radio frequency (RF) point-by-point ablations. Cryoballoon AF ablations were not evaluated at all in the majority of these studies [2,3,7,8] and encompassed a small minority (8%) of ablations in others [9]. Given the growing use of cryoballoon AF ablation in the last decade, the freezing-induced sealing of the cryoballoon surface with PV ostia, whereby the freezing is not only used to create the ablation but also to enforce a tight contact between balloon and ostia [12], and its ability to create circumferential trans-mural PV scar in animal studies [13,14], we sought to evaluate cryoballoon AF ablation’s ability to create circumferential PV fibrosis using post-ablation LGE MRI and its correlation with AF recurrence. Notably, although few prior studies have evaluated MRI post cryoballoon AF ablation [15,16,17,18], only a single cryoballoon study evaluated both pre- and post-ablation MRI to reflect ablation-induced PV lesions [15]. Furthermore, in contrast with prior studies methodology of assessing post-ablation MRI PV gaps, we used a new methodology whereby complete and sub-complete circumferential PV ostial fibrosis were grouped together; thus, only major PV ostial gaps were considered (see methods). 

## 2. Materials and Methods

### 2.1. Study Design and Population

This prospective study included patients planned for first AF ablation due to symptomatic drug-refractory AF. All patients underwent pre- and post-ablation MRI to assess baseline and ablation-induced fibrosis, respectively. To simplify the detection of ablation-induced atrial fibrosis, we selected a priori patients without significant structural heart disease, anticipating they would have minimal atrial fibrosis at baseline. Significant structural disease included any clinical or imaging evidence of active ischemia, LV dysfunction defined by LV ejection fraction < 50%, any valvular regurgitation/stenosis ≥ moderate severity, pulmonary hypertension (TIG > 45 mmHg), or LA anteroposterior diameter > 50 mm on baseline TTE. 

#### 2.1.1. Study Inclusion Criteria

− Age 18–75;
− Symptomatic AF, refractory to at least one anti-arrhythmic medication.

#### 2.1.2. Study Exclusion Criteria

− Significant structural heart disease as defined above, except of hypertension (HTN);
− Prior heart surgery or prior ablation involving the left atrium;
− Pregnant women;
− Inability to undergo cardiac MRI with gadolinium injection either due to claustrophobia or significant renal disease (GFR < 30 mL/min).

#### 2.1.3. Study Endpoints

Our aim was to evaluate cryoballoon AF ablation’s ability to create circumferential PV fibrosis using post-ablation LGE MRI and its correlation with AF recurrence. 

### 2.2. Cryoballoon AF Ablation Procedure

All study patients underwent first AF ablation using the second-generation 28 mm cryoballoon (Arctic Front Advance Pro, Medtronic, Minneapolis, MN, USA) consisting of PV electrical isolation per se, in a dedicated protocol. Prior to ablation, all patients underwent cardiac CT to evaluate LA and PV anatomy and rule out LA thrombi. The ablation procedure started with a trans-septal puncture using a Brockenbrough needle under ICE guidance, heparin infusion thereafter keeping an ACT > 300 s, LA venography to define PV anatomy, cannulation of each PV with the cryo “achieve” catheter, inflating the cryoballoon and advancing it to occlude the PV ostium (confirmed by contrast injection), and 3 min’ cryoballoon application in each PV. Whenever PV potentials were seen, the time to isolation was recorded, and if >90 s, a second cryoballoon application was performed for another 2 min. Notably, left common PVs were marked but counted as two PVs, namely LIPV and LSPV, with a cryoballoon application in each of these main branches. After cryoballoon application in all PVs, PVI was confirmed by pacing within each PV and outside the PV in a nearby location (LAA or distal CS for left-sided PV and SVC for right-sided ones) to confirm exit and entrance block, respectively. Thereafter, heparin was stopped, anticoagulation reversed with protamine, and catheters pulled out with manual pressure.

### 2.3. Cardiac MRI Protocol

All patients underwent pre- and post-ablation MRI using identical protocol. Pre-ablation MRI was performed 1 month prior to the index AF ablation, and the post-ablation MRI was performed 3–6 months post index ablation. All MRIs were performed using 1.5 T scanner (Aera, Siemens Healthcare, Germany) with an 18-channel body coil. After scout sequences, cine images in short-axis oblique and long-axis views (two-chamber, three-chamber, and four-chamber) were acquired. Then, PSIR LGE images in the same anatomical views were acquired ten minutes following a bolus injection of 0.2 mmol/kg of gadoterate meglumine (Dotarem; Gadoteric acid—Gadoterate melgumine, France). The 3D LA LGE scan was performed 15 min after the injection of the gadolinium and consisted of a free-breathing respiration-navigator-gated 3D FLASH sequence with FatSat and ECG-gating. Low flip angles were applied to reduce saturation effects with short repetition times. The receiver bandwidth was 300 KHz/pixel, and the slice thickness 1.5 mm pixel spacing was 0.7 × 0.7 mm. Inversion time was set according to a scout sequence in a mid-ventricular image. No parallel imaging was used.

### 2.4. MRI Definitions and Categorization of Ablation-Induced PV Ostial Fibrosis

**Figure 1:**
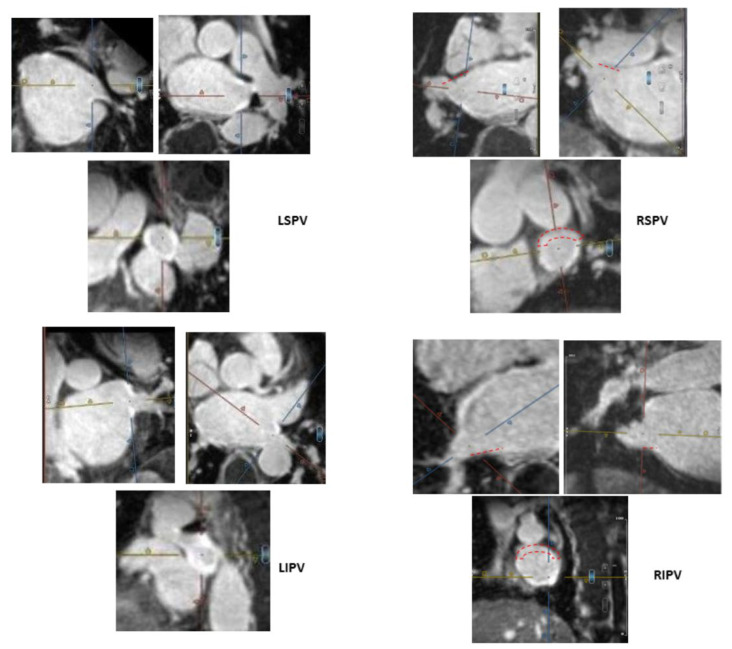
Cross-sectional short-axis views of PVs on post-ablation LGE MRI using the “double oblique” technique. For each PV, two orthogonal plane images are used to create its circular cross-sectional short-axis view. (PV are centered between the MRI orthogonal lines.) Each PV ostium was divided into four segments: anterior, posterior, roof, and floor. Based on the cross-sectional views of PV ostia, ablation-induced PV ostial fibrosis are categorized as complete circumferential fibrosis, sub-complete circumferential fibrosis with single “minor” gap defined as LGE gap ≤ 1/3 PV segment length, partial fibrosis with major gaps defined by as LGE gap > 1/3 segment length, or absent PV fibrosis. In this patient, both left PVs are fully encircled by LGE enhancement (seen as bold white lines), defined as complete PV circumferential fibrosis, while both right PVs have major gaps (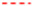) and thus are defined as partial incomplete circumferential PV ostial fibrosis. The right PV gaps are marked (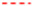) both on the orthogonal plane images and the cross-sectional view.

Ablation-induced fibrosis in the LA body and PV ostia were evaluated by two experienced readers (A.W. and M.R.) in the following manner. The 3D LA inversion recovery LGE scan was uploaded to an MPR viewer (cvi42^®^, Circle Cardiovascular Imaging Inc, Calgary, Canada). A cross-sectional short-axis view of each PV ostium was acquired using two orthogonal long-axis views (“double oblique technique”; Figure 1). The LA body LGE extent was assessed quantitatively by ADAS software 3D LA model (ADAS 3D^®^, Barcelona, Spain), while the PV ostia LGE extent was assessed semi-quantitatively, both by exploration of each PV ostium cross-sectional short-axis view (original images analysis) and assisted by visual analysis of the computerized ADAS 3D LA model, as previously described by others [15,16,19]. This software automatically calculates the image intensity ratio (IIR) of LGE in LA wall and PV ostia relative to the LA blood pool, thus standardizing the LGE intensities in all patients in a reproducible way [20]. LGE IIRs of <1.2 and >1.32 were shown to represent normal LA tissue and dense LA scar, respectively [20,21]. The assessment of PV ostial fibrosis was primarily based on a semi-quantitative analysis of the original MRI short-axis PV ostia views and only supplemented by the computerized ADAS software analysis (to minimize impact of potential software pitfalls).

Ablation-induced PV ostial LGE was categorized based on the presence and extent of LGE gaps. Each PV ostium was divided into four segments: roof, floor, anterior, and posterior. LGE gap width ≤1/3 segment length was defined as a minor gap, while LGE gap >1/3 segment length was defined as a major gap (Figure 1). Accordingly, ablation-induced PV ostial fibrosis was categorized as follows:

− Complete—circumferential PV ostial LGE without gaps;
− Sub-complete—an almost circumferential LGE around PV ostium with the presence of a minor LGE gap only;
− Partial—incomplete circumferential LGE around PV ostium due to the presence of a major LGE gap;
− Absent PV fibrosis—no LGE around PV ostium.

Given our semi-quantitative assessment of PV ostial LGE extent and the challenge present at times in discerning between a complete and sub-complete PV ostial fibrosis, we decided to consider both as a single group defined as “circumferential” PV ostial fibrosis (defined by the absence of major PV gaps). In all patients, PV ostial fibrosis categorization by LGE-MRI was made by a consensus of the two above-mentioned readers.

Atrial arrhythmia recurrence was monitored via multiple ECG, 24-h Holters on routine EP clinic visits at 1, 3, 6, and 12 months post ablation and a monthly cardiac monitoring device (“Heart One” event recorder; Monitor Company, Israel) at 6 and 12 months post ablation for all patients. The study was approved by Shaare Zedek Hospital Helsinki Committee, and all study participants signed an informed consent.

### 2.5. Statistics

Categorical variables were summarized as frequency and percentage. Continuous variables were evaluated for normal distribution using histogram and Q-Q plot and were reported as mean and standard deviation or as median and inter-quartile range. Statistical significance calculated via chi-square test for categorical variables and via *t*-test for continuous variables (*p* < 0.05 considered significant). Kaplan–Meier (KM) survival curves were performed for study main outcome regarding correlation of post-ablation PV fibrosis on MRI and AF recurrence using log-rank analysis.

SPSS software was used for all statistical analyses (IBM SPSS statistics for windows, version 27, IBM Corp, Armonk, NY, USA, 2020).

## 3. Results

Twenty-two consecutive patients with symptomatic drug-refractory AF and no significant cardiac structural abnormalities and who were planned for first AF ablation were recruited for the study. All patients underwent baseline pre-ablation MRI, AF cryoballoon ablation (“index” ablation), and a post-ablation MRI 3–6 months after the index ablation, with an average of 4±1.1 months after the index ablation procedure. There were 3/22 patients with low-quality post-ablation LGE MRI images precluding accurate evaluation of post-ablation PV fibrosis; thus, these patients were excluded. Accordingly, our study included 19 patients with 80 PVs (including four patients with five PVs due to the presence of a right middle PV). Their age was 56 ± 11, and 9/19 (47.3%) were females, with paroxysmal and persistent AF in 17/19 (89.5%) and 2/19 (10.5%) patients, respectively. Medical history included hypertension among 11/19 patients (57.9%), diabetes in 3/19 patients (15.8%), and chronic renal failure (creatinine > 1.4 mg%) and diastolic heart failure both occurring among 1/19 patients (5.2%). All patients had normal LV function, no significant valvular abnormalities, and LA diameter of 39.5 ± 4.3 mm on TTE (Table 1). Baseline MRI revealed LGE covering 6.5 ± 4% of LA body surface area. Among 17/19 (89.5%) patients, baseline LA fibrosis was minimal (≤10%) and compatible with grade I LA fibrosis by Utah Classification [2,4]. There were 2/19 patients with baseline LA fibrosis covering 11–20% of LA surface area, compatible with grade II LA fibrosis by Utah Classification. All patients underwent a successful cryoballoon PVI, with successful electrical isolation of all PVs confirmed by the presence of an exit and entrance block.

**Figure 2:**
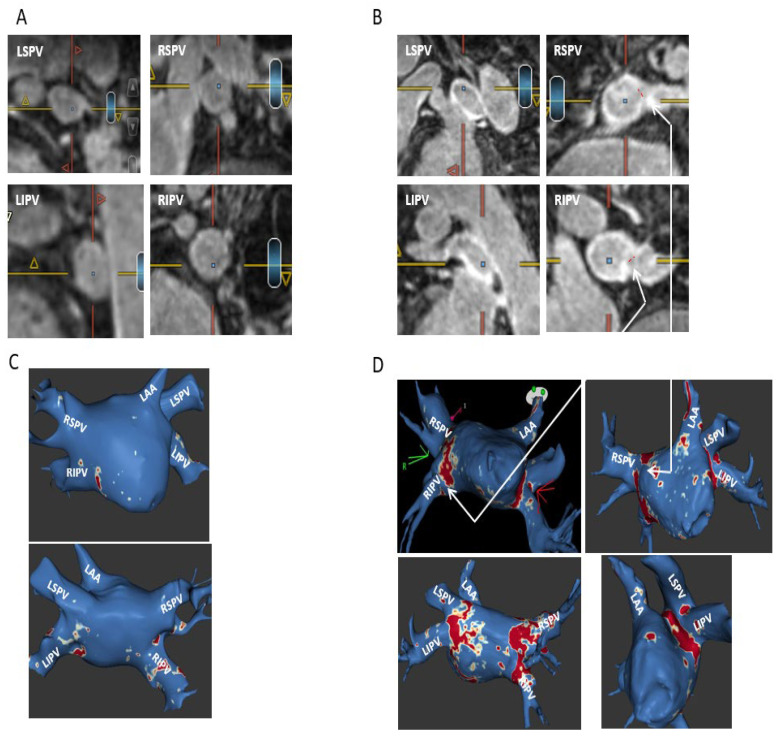
A typical patient with cryoballoon-induced complete/sub-complete circumferential fibrosis in all PVs. Shown are pre-ablation (**A**) and post-ablation (**B**) cross-sectional short-axis views of the four PVs revealing minimal PV ostial fibrosis at baseline, while at post AF ablation, there is a complete circumferential fibrosis around both left PVs and sub-complete circumferential fibrosis around both right PVs, with minimal gaps in the anterior segments of both veins (red dottedlines). (**C**,**D**) Cor-responding ADAS 3D LGE model images pre (**C**) and post (**D**) cryoballoon AF ablation showing minimal LA fibrosis at baseline, post-ablation complete circumferential fibrosis around both left PVs without any gaps, and sub-complete circumferential fibrosis around both right PVs due to minimal gaps at their anterior segments, corresponding with the original images analyses. The post-ablation fibrotic areas on ADAS are colored red, while PV gaps are seen as blue color. The arrows show the matching of the minor PV gaps around both right PVs between the original MRI cross-sectional views and the ADAS model images.

**Figure 3:**
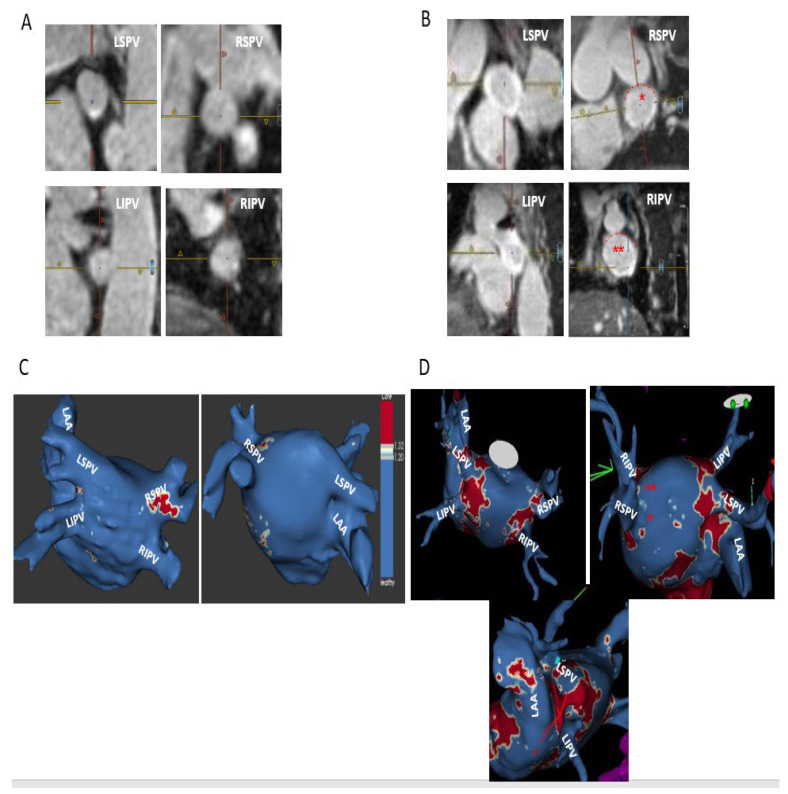
A typical patient with cryoballoon-induced partial incomplete circumferential fibrosis in both right PVs. Shown are pre-ablation (**A**) and post-ablation (**B**) cross-sectional short-axis views of the four PVs revealing minimal PV ostial fibrosis at baseline, while at post AF ablation, there is a complete circumferential fibrosis around both left PVs and partial incomplete circumferential fibrosis around both right PVs with major gaps in the roof and anterior segments of both veins (red dotted lines). (**C**,**D**) Corresponding ADAS 3D LGE model images pre (**C**) and post (**D**) cryoballoon AF ablation, showing minimal fibrosis at baseline (**C**). Post-ablation MRI (**D**) reveals a complete circumferential fibrosis around both left PVs (including the carinal view) and partial circumferential fibrosis of both right PVs due to major gaps in the anterior and roof segments of both RSPV (*) and RIPV (**), corresponding and matching with the gaps seen on the “raw data” short-axis views. Post-ablation fibrotic areas on ADAS are colored red. Notably, Figure 1 and Figure 3 both describe the same patient.

Post-ablation MRI revealed complete/sub-complete circumferential PV ostial fibrosis among 63/80 (78.8%) PVs, partial PV ostial fibrosis in 15/80 (18.7%) PVs, and absent PV ostial fibrosis in a 2/80 (2.5%) PVs. Partial PV ostial fibrosis occurred in the following PVs (number of PVs with partial fibrosis/total number of these PVs in the study): LSPV (1/19), LIPV (3/19), RIPV (4/19), RSPV (6/19), and RMPV (1/4). The two PVs with absent ostial fibrosis post-ablation were LSPV (*n* = 1) and LIPV (*n* = 1). There were 10/19 (52.6%) patients in whom all PVs had complete/sub-complete ablation-induced circumferential ostial fibrosis (hence no major PV ostial gaps) and 9/19 (47.4%) of patients in whom one or more PVs had partial circumferential or absent ostial fibrosis, with one or more major PV ostial gaps (Figure 1). Shown are a typical patient with complete/sub-complete circumferential fibrosis around all PV ostia after cryoballoon AF ablation (Figure 2) and a patient in whom both right PVs had partial PV ostial fibrosis after ablation due to the presence of major gaps in these PVs (Figure 3).

**Figure 4:**
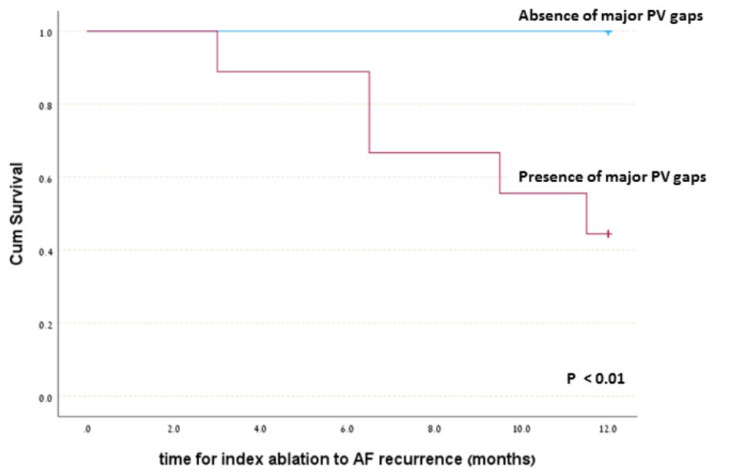
Kaplan–Meier (KM) survival without AF recurrence analysis according to presence or absence of major PV ostial gaps. The analysis shows absence of AF recurrence among the group without major PV gaps and a significant AF recurrence among the groups with major PV gaps. This KM analysis reinforces the significant correlation between presence of major PV ostial gaps and AF recurrence.

Atrial fibrillation recurred in 5/19 (26.3%) patients within one year, all of which had major PV ostial gaps on post-ablation MRI and hence are categorized as partial PV ostial fibrosis. There were no significant differences between the subgroups with and without AF recurrence regarding patients’ age, gender, background illness, and various known potential AF triggers including obstructive sleep apnea, hypertension, diabetes mellitus, chronic renal failure, diastolic heart failure, ischemic heart disease, AF duration (time period from first AF diagnosis to index ablation), anti-arrhythmic drugs (AAD) used after the index ablation, valvular disease, and LA size (Table 1). Nevertheless, significant association was found between major PV gaps on post-ablation MRI and AF recurrence. Analysis of AF recurrence according to index ablation-induced circumferential PV fibrosis revealed no evidence of AF recurrence in all 10 patients in whom all PVs were found to have a complete/sub-complete circumferential ostial fibrosis (i.e., without any major PV ostial gaps), while AF recurrence was found among 5/9 patients in whom a partial ablation-induced PV ostial fibrosis (i.e., presence of major PV gaps) was found around one or more of their PVs on post-ablation MRI. Accordingly, a significant correlation was found between the presence of PV major gaps seen on post-ablation MRI and one-year AF recurrence (5/9 versus 0/10; *p* < 0.01). This association was also shown via Kaplan–Meier survival without AF recurrence curve, revealing a significantly increased rate of AF recurrence among the group with major PV gaps (Figure 4, *p* < 0.01). Based on these results, the negative and positive predictive value of major PV gaps seen on post-ablation MRI for AF recurrence were 100% and 55.6%, respectively (Table 2).

**Table 2:** Sensitivity, specificity, and negative and positive prediction values of major PV gaps for AF recurrence after AF ablation.

	AF Recurrence (*N* = 5)	No AF Recurrence (*N* = 14)	Total
Patients with major PV gap in one or more PVs	5 (True Positive)	4 (False Positive)	9
Patients without major PV gap in any PV	0 (False Negative)	10 (True Negative)	10

Sensitivity = 100% Specificity = 71.4% Positive predictive Value (PPV) = 55.6% Negative Predictive Value (NPV) = 100%Sensitivity = TP/(Tp + FN) Specificity = TN/(TN + FP) PPV = TP/(Tp + FP) NPV = Tn/(FN + TN)

The association of major PV gaps with AF recurrence was further evaluated by comparing the percent of PVs with circumferential fibrosis between the groups with and without AF recurrence. In our study, there were 5 patients with 21 PVs in whom AF recurred within a year and 14 patients with 59 PVs in whom AF did not recur within a year. There was a significantly higher percent of PVs with circumferential PV ostial fibrosis (without major gaps) among the group without AF recurrence as compared to the group with AF recurrence (51/59 (86.5%) versus 12/21 (57.1%); *p* < 0.01).

**Figure 5:**
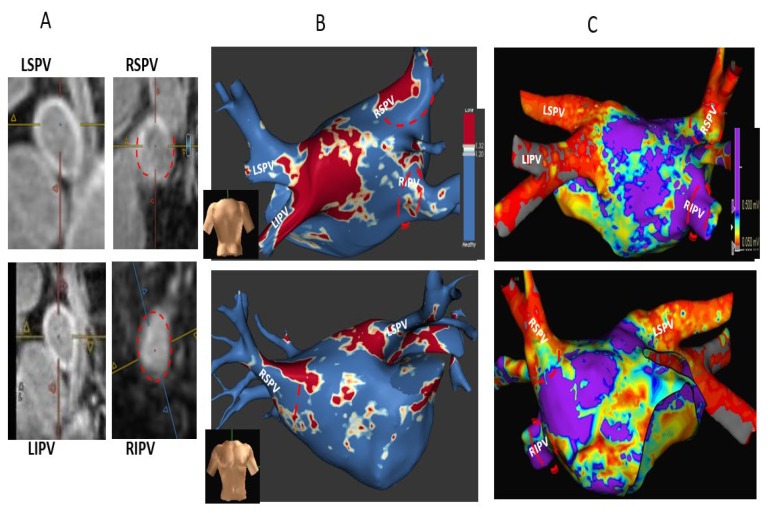
Correlation of post-index-ablation MRI to redo procedure electro-anatomic mapping (EAM): Major PV gaps around both right PVs (upper-posterior views, lower-anterior views). Post-index-ablation MRI cross-sectional short-axis views (“raw data” images) (**A**), post-index-ablation MRI ADAS 3D model (**B**), and redo procedure EAM (Ensite Precision system) (**C**) all show complete circumferential PV ostial fibrosis around the main branches of a left common PV (marked as LSPV and LIPV); presence of major gaps in RSPV anterior, inferior, and posterior walls (marked by red dotted lines); and almost absent ostial fibrosis around RIPV (red dotted lines). Good correlation was found between post-ablation MRI and redo EAM regarding PV ostial scar and major gap locations (marked by red dotted lines in both MRI and EAM images).

**Figure 6:**
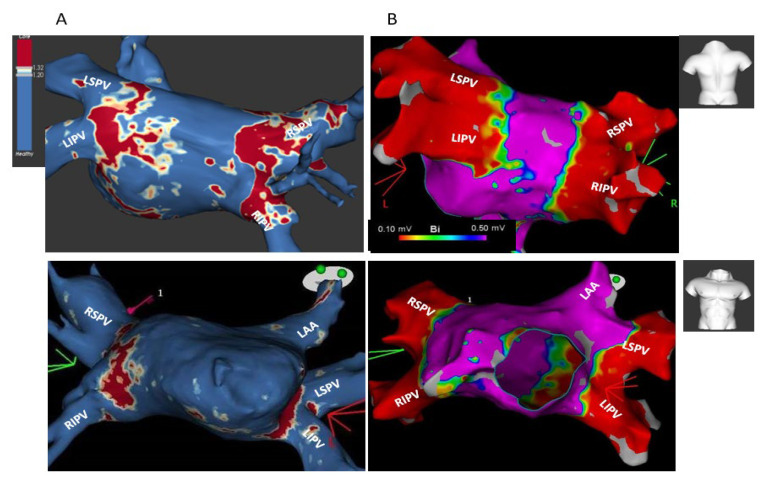
Correlation of post-index-ablation MRI to redo procedure electro-anatomic mapping (EAM): Circumferential PV ostial fibrosis around all PVs (upper-posterior views, lower-anterior views). Post-index-ablation MRI (**A**) shows a complete circumferential PV fibrosis around both left PVs and sub-complete circumferential fibrosis around both right PVs. Due to newly documented atypical AFL, the patient underwent redo ablation, which started with LA EAM (Carto system), revealing circumferential scars around all PV ostia (**B**). A good correlation found between post-index-ablation MRI and redo procedure EAM shown. Notably, Figure 2 and Figure 6 both describe the same patient.

Redo ablations were performed in 4/5 patients with AF recurrence and in 1 patient who was free of AF but had new documentation of atypical atrial flutter. Redo ablations were started by LA EAM to locate PV ostial gaps. Comparison of post-index-ablation MRI and redo EAM revealed a fair match between these two imaging modalities, showing major gaps among the same PVs with similar gap location within each PV. Minor gaps on MRI correlated with complete scar on EAM. A typical example is shown in Figure 5, revealing circumferential PV ostial fibrosis in a left common PV, major gaps in RSPV anterior and inferior walls, and absent ostial fibrosis in RIPV in both post-index-ablation MRI (Figure 5A,B, corresponding to MRI cross-sectional views and ADAS 3D model) and redo procedure EAM (Figure 5C). Figure 6 shows an example of circumferential PV fibrosis among all PVs in a patient with atypical atrial flutter who was free from AF. A complete circumferential fibrosis around both left PVs and sub-complete circumferential fibrosis around both right PVs was seen in post-index-ablation MRI (Figure 6A), correlating with circumferential scars around all PVs on the redo procedure EAM (Figure 6B). Notably, all PVs in this patient were electrically isolated.

## 4. Discussion

Our prospective study included 19 consecutive patients with drug-refractory AF and structurally normal hearts who underwent a successful cryoballoon electrical isolation of all PVs. All patients underwent baseline pre-ablation MRI and a post-ablation MRI according to the same protocol. On baseline MRI, 90% of patients had minimal (<10%) LA fibrosis, as expected by the absence of significant structural heart disease, according to our study inclusion criteria. Post-ablation MRI, performed 3–6 months post index cryoballoon AF ablation, revealed high prevalence (78.8%) of complete or sub-complete circumferential PV ostial fibrosis with more than half of patients (10/19; 52.6%), in whom all PVs had circumferential ostial fibrosis without major gaps. During one-year follow-up post index ablation, there were 5/19 (26.3%) patients with AF recurrence, all of which had major PV gaps on post-ablation MRI, while no AF recurred among the 10 patients with circumferential fibrosis of all PVs. Thus, a significant association was found between the presence of major gaps on post-ablation MRI and clinical AF recurrence (*p* < 0.01).

Our study’s first endpoint was the relatively high percent of complete or sub-complete circumferential PV ostial fibrosis, occurring among 78.8% of ablated PVs, resulting in 52.6% of patients in whom all PVs were encircled by fibrosis. This result is apparently higher than the incidence of RF ablation-induced PV circumferential fibrosis reported in many prior MRI studies [7,8,9,10,19]. In these studies, only 4% (4/94) [19], 7% (12/177) [9], or 12.5% (7/56) [7] of patients had circumferential fibrosis around all PVs on MRI performed 3–15 months post RF ablation. As of today, only few prior MRI studies have evaluated cryoballoon-induced PV fibrosis [15,16,17], among which only one study included both pre- and post-ablation MRI [15]. These few cryoballoon studies also showed only a small minority of patients having circumferential fibrosis around all PVs on post-cryoballoon-ablation MRI [15,16,17]. Importantly, the grouping together of complete and sub-complete circumferential PV fibrosis in our study while “ignoring” minor gaps probably led to over-estimation of the true prevalence of complete circumferential PV fibrosis. Thus, we could not compare our result to most prior MRI studies done after RF or cryoballoon AF ablations, where minor gaps were considered as well. Nevertheless, in comparison with prior studies, we suggest that our lenient semi-quantitative approach for assessing post-ablation PV ostial fibrosis is advantageous from a clinical perspective given MRI limitation in precisely identifying post-ablation PV gaps. This limitation is suggested by the following:

1. In many prior MRI studies, both high LGE intensities (corresponding to dense scar) and intermediate intensities (corresponding to interstitial fibrosis) were considered as scar for the sake of gap analysis to avoid gap over-estimation, suggesting that MRI has limited ability to differentiate dense scar from interstitial fibrosis and has a tendency for over-estimation of PV gaps [7,15,16,19];
2. Multiple studies show the strongest predictor for AF recurrence after ablation is the “relative gap length”, defined as total length of all gaps in each PV divided by PV perimeter, rather than the number of gaps or each specific gap length [15,16,19];
3. Changing gap definition from 3 to 5 mm on post-ablation MRI did not change AF recurrence results [19];
4. Animal studies showing conduction block persisting in the presence of post-ablation MRI gaps of up to 4 mm [22].

The above findings all suggest post-ablation PV gap analysis via MRI should not assess every mm of gap but should rather focus on semi-quantitative assessment of PV “fibrotic extent”, speaking in favor of grouping complete and sub-complete PV fibrosis (with minor gaps) together and differentiating these from partial PV fibrosis, which is defined by the presence of major gaps. It is worth noting that most of the major PV ostial gaps revealed in our study involved the right PVs, which is in line with prior studies [15,16,23].

Using our lenient approach to evaluate post-ablation gaps on MRI, we could also show for the first time a significant correlation between the presence of circumferential PV ostial fibrosis around all PVs on post-ablation MRI and the absence of AF recurrence (*p* < 0.01), which is our study’s second and most important endpoint. Indeed, no AF recurred among patients in whom all PVs were completely or sub-completely encircled by fibrosis on post-ablation MRI (n = 10), suggesting that minor gaps on MRI are still associated with absence of AF recurrence. Prior studies have mostly shown a poor correlation between PV fibrosis on post-ablation MRI and AF recurrence [8,9,10,11]. We suggest that the absence of significant correlation in most prior studies was related to their strict definition of post-ablation PV ostial fibrosis, where every gap was considered, leading to a small percentage of patients with circumferential fibrosis around all PVs after AF ablation [2,8,9,10,19]. Thus, it is conceivable that our semi-quantitative assessment of PV ostial fibrotic extent, “ignoring” minor PV gaps, highlighted the association between post-ablation PV fibrosis and AF recurrence. Accordingly, we suggest that our semi-quantitative MRI-LGE analysis might be a more clinically relevant approach compared with prior apparently “precise” analytic methods, where every mm gap on MRI was considered as a true PV gap.

There were five patients in our study who underwent redo ablation, revealing a fair match between the findings of the post-index-ablation MRI and redo procedure EAM regarding PV major gap locations and the presence or absence of circumferential PV ostial fibrosis. Interestingly, sub-complete circumferential fibrosis (due to minor gaps) on post-ablation MRI correlated with a compete scar on redo EAM, suggesting once more that sub-complete and complete PV ostial fibrosis on MRI should be analyzed as one (“ignoring” minor PV gaps), as done in our study. To date, MRI reliability and accuracy in detecting post-ablation PV gaps is under major debate. Some studies suggest it to be accurate and capable of guiding redo ablations [5], while others suggest it to be inaccurate and poorly correlated with redo procedure EAM [6,7,8]. Given the very few patients in whom we could test this correlation in our study, we are cautious in judging MRI performance in this issue. Nevertheless, this was not the aim of our study, and we intend to present the correlation of post-ablation MRI and redo procedure EAM in a larger study that is currently ongoing.

The relationship between presence of circumferential fibrosis of all PVs on post-ablation MRI and AF recurrence, as found in our study, suggests post-ablation MRI to have an excellent negative predictive value (approaching 100%) and a modest positive predictive value (55%) for AF recurrence. The modest positive predictive value is expected since PV ostial gaps were shown to be present in many patients after AF ablation regardless of the presence or absence of AF recurrence [15,16,17,19]. This was confirmed by a few studies where an invasive EAM was routinely performed after AF ablation, revealing PV gaps among 70% and 90% of patients without AF recurrence at 3 and 12 months post ablation, respectively [24,25]. Thus, the presence of post-ablation gap does not necessarily imply AF recurrence. Nevertheless, the high negative predictive value of MRI suggests it to be a useful tool to predict freedom from AF recurrence in patients with structurally normal hearts who do not have major PV gaps on post-ablation MRI. Accordingly, a possible clinical application of post-ablation MRI would be to serve as a non-invasive tool to appreciate AF ablation success and guide future follow-up as well as assisting in the planning of redo ablation procedure in case of recurrent AF. For example, the presence of major PV ostial gaps on MRI, performed in a timely manner within 3–6 months post AF ablation, would warrant a more vigorous follow-up for AF recurrence. Future studies may shed light on the ability of these MRI PV gaps to guide redo ablations. Furthermore, in the case of recurrent AF in the absence of PV gaps on post-ablation MRI, one would need to plan redo ablation procedure including search for non-PV triggers (anticipating that all PV would be isolated on the redo procedure). Another clinical application would be to evaluate and compare new ablation techniques and different energy sources (for example, to compare cryoballoon with the newly introduced RF balloon in creating PV circumferential ostial lesions).

### Limitations

Our study has several limitations, including the following: (1) There was a small number of patients, limiting generalization of our study results and preventing us from drawing firm conclusions. Indeed, all study results need to be confirmed in a larger population. (2) The study population included a relatively healthy population without significant structural heart disease, mostly with paroxysmal AF, with minimal baseline LA fibrosis on pre-ablation MRI. Thus, our results are limited to this population and should be tested among sicker patients with persistent AF and increased baseline LA fibrosis; notably, in this study, we selected a relatively healthy AF population with minimal baseline LA fibrosis to enhance our ability to detect ablation-induced LA fibrosis. (3) There were few patients only who underwent redo ablation, in whom we could compare post-index-ablation MRI to redo procedure EAM. Nevertheless, we stress that this was not the aim of our study, which was intended to evaluate cryoballoon PVI ability to create circumferential PV fibrosis and its correlation to AF recurrence by using a new relatively novel semi-quantitative approach, which was shown here to be very relevant clinically. (4) All study patients underwent post-ablation MRI 3–6 months post ablation. Since a decreased reliability of late post-ablation MRI performed > 12 months after ablation has been suggested [21], our study results do not necessarily apply to patients in whom post-ablation MRI was performed at later times.

## 5. Conclusions

Cryoballoon AF ablation among patients with structurally normal hearts results in a complete or sub-complete circumferential PV ostial fibrosis in majority (78.8%) of electrically isolated PVs, as assessed by the comparison of post- to pre-ablation LGE-MRI, using a new clinically relevant semi-quantitative LGE analysis method. In over half of the patients, a circumferential fibrosis was found around all PVs. A significant correlation was found between the absence of major PV gaps on post-ablation MRI and freedom from AF recurrence, suggesting that post-ablation MRI might have a reasonably negative predictive value for AF recurrence in cases where all PVs have circumferential PV ostial fibrosis.

## Figures and Tables

**Table 1:** Patient data comparison between subgroups with and without one-year AF recurrence.

	Total *N* = 19	No AF Recurrence *N* = 14	AF Recurrence *N* = 5	*p*
Age	55.2 ± 12	54 ± 1	59 ± 9	0.6
Female	10/19 (52.6%)	7/14 (50%)	3/5 (60%)	0.9
BMI	26 ± 2.8	25.9 ± 3	26 ± 1.8	0.9
HTN	11/19 (57.9%)	7/14 (50%)	4/5 (80%)	0.3
DM	3/19 (15.8%)	2/14 (14.3%)	1/5 (20%)	0.9
Chronic renal failure	1/19 (5.2%)	1/14 (7.2%)	0/5 (0%)	0.8
Heart failure	1/19 (5.2%)	1/14 (7.2%)	0/5 (0%)	0.8
Ischemic heart disease	0/19 (0%)	0/14 (0%)	0/5 (0%)	1
Obstructive sleep apnea	3/19 (15.8%)	2/14 (14.3%)	1/5 (20%)	0.9
Persistent AF	2/19 (10.5%)	2/14 (14.3%)	0/5 (0%)	0.5
AF duration (months) *	37 ± 31	41 ± 33	36 ± 30	0.7
AAD use post index ablation **	11/19 (57.9%)	7/14 (50%)	4/5 (80%)	0.3
LV dysfunction	0/19 (0%)	0/19	0/5	1
Mitral regurgitation mild/mild-moderate	14/19 (73.7%)	4/5 (80%)	11/14 (78.5%)	0.9
Tricuspid regurgitation mild/mild-moderate	13/19 (68.5%)	3/5 (60%)	10/14 (71.5%)	0.6
Tricuspid gradient (mmHg)	21.5 ± 7.5	23 ± 4	21 ± 9	0.5
LA diameter (mm) ***	39.5 ± 4.3	38 ± 3.8	42 ± 5.5	0.1
Partial PV fibrosis (major gap) on post-ablation MRI	9/19 (47.4%)	4/14 (28.5%)	5/5 (100%)	0.01

* AF duration defined as time period from first AF documentation to “index” ablation. Does not imply AF persistence but rather the time period within which episodic AF occurred. ** AAD, anti-arrhythmic drug. *** LA antero-posterior diameter (mm).

## Data Availability

Data unavailable due to ethical restrictions but can be given anonymously by request.
